# Untreated primary hypothyroidism with simultaneous rhabdomyolysis, pericardial effusion, and sudden sensorineural hearing loss: a case report

**DOI:** 10.1186/s12902-019-0379-y

**Published:** 2019-05-22

**Authors:** Chung Gyo Seo, Kyoung Jin Kim, Euyhyun Park, Nam Hoon Kim, Joo Hyung Kim, Hee Young Kim, Sin Gon Kim, Kyeong Jin Kim

**Affiliations:** 1 Division of Endocrinology and Metabolism, Department of Internal Medicine, Korea University Anam Hospital, Korea University College of Medicine, 73 Inchon-ro, Seongbuk-gu, Seoul, 02841 Republic of Korea; 2 Department of Otolaryngology-Head and Neck Surgery, Korea University Anam Hospital, Korea University College of Medicine, Seoul, South Korea

**Keywords:** Hypothyroidism, Rhabdomyolysis, Pericardial effusion, Hearing loss

## Abstract

**Background:**

Hypothyroidism, one of the prevalent endocrine disorders worldwide, has a broad spectrum of clinical manifestations, from an asymptomatic condition to myxedema coma. Although the majority of patients with hypothyroidism have minor clinical symptoms, which are recovered with levothyroxine treatment, some patients occasionally do experience fatal complications. Here we report, for the first time, the case of a patient who had hypothyroidism with simultaneous occurrence of rhabdomyolysis with acute kidney injury, moderate pericardial effusion, and sudden sensorineural hearing loss.

**Case presentation:**

A 57-year-old man with a previous history of dyslipidemia and untreated hypothyroidism was admitted to the hospital due to shortness of breath, lethargy, lower extremity discomfort, and unilateral hearing loss. Laboratory results revealed rhabdomyolysis with acute kidney injury and severe hypothyroidism. We detected cardiomegaly without lung parenchymal infiltration on chest radiography and moderate pericardial effusion on transthoracic echocardiography. We performed pure tone audiometry and identified profound unilateral sensorineural hearing loss. Aggressive fluid resuscitation, levothyroxine treatment, and systemic and intratympanic steroid therapy alleviated the patient’s severe hypothyroidism, rhabdomyolysis, and pericardial effusion; however, sensorineural hearing loss was not fully recovered.

**Conclusions:**

Early recognition of life-threatening complications is important in patients with severe hypothyroidism to prevent adverse outcomes. This case suggests that hypothyroidism should be considered in patients who have rhabdomyolysis with acute kidney disease and pericardial effusion. Moreover, sudden sensorineural hearing loss should be kept in mind as a rare complication of hypothyroidism.

## Background

Hypothyroidism, characterized by elevated level of serum thyroid stimulating hormone (TSH) with lower or normal free thyroxine (fT4), is predominant worldwide, particularly in iodine-deficient regions [1–3]. Previous studies have reported that the general prevalence of hypothyroidism is 5–10% in women and 1–3% in men [4–6]. Clinical manifestations of hypothyroidism vary widely from asymptomatic or subclinical conditions to overt state of myxedema coma or multiorgan failure, depending on the age at diagnosis, the duration and severity of thyroid hormone deficiency. The most common symptoms of hypothyroidism are fatigue, cold sensitivity, constipation, dry skin, and weight gain. Carpal tunnel syndrome, voice change, and myopathy are less common signs and rhabdomyolysis, pericardial effusion, and sudden hearing loss are rare complications of hypothyroidism [1, 7]. Although previous studies have reported a few cases of these rare complications, such as rhabdomyolysis or pericardial effusion due to overt hypothyroidism [8–10], there are few reports of simultaneous occurrence of rare complications caused by primary hypothyroidism. Of particular note is that early recognition of combined complications induced by hypothyroidism is essential to treat patients who can recover with simple levothyroxine treatment.

In this regard, we describe an unusual case of severe hypothyroidism accompanied by rare complications of rhabdomyolysis with acute renal injury, pericardial effusion, and sudden sensorineural hearing loss at the same time.

## Case presentation

A 57-year-old man presented to the emergency room with a several-day history of shortness of breath, nausea, dizziness, bilateral limb discomfort, and unilateral hearing loss. He also complained of cold intolerance and sluggish speech and movement that started a few weeks earlier, as well as 2-kg weight gain over the past year. He started rosuvastatin (5 mg, once daily) a year ago, with no dose adjustment; the patient was diagnosed with hypothyroidism at the same time. He was recommended levothyroxine treatment but did not initiate treatment because he had no related symptoms or discomfort in his daily life.

General physical examination revealed dry skin, neck vein distension, nontender diffuse goiter around the neck, and myxedema with puffy face, bilateral periorbital and lower extremities edema. His pulse rate was 52 beats/minute, blood pressure was 114/82 mmHg, respiratory rate was 20 breaths/minute, and his body temperature was 37.1 °C. Muffled heart sounds without fine crackle were also detected.

**Fig. 1:**
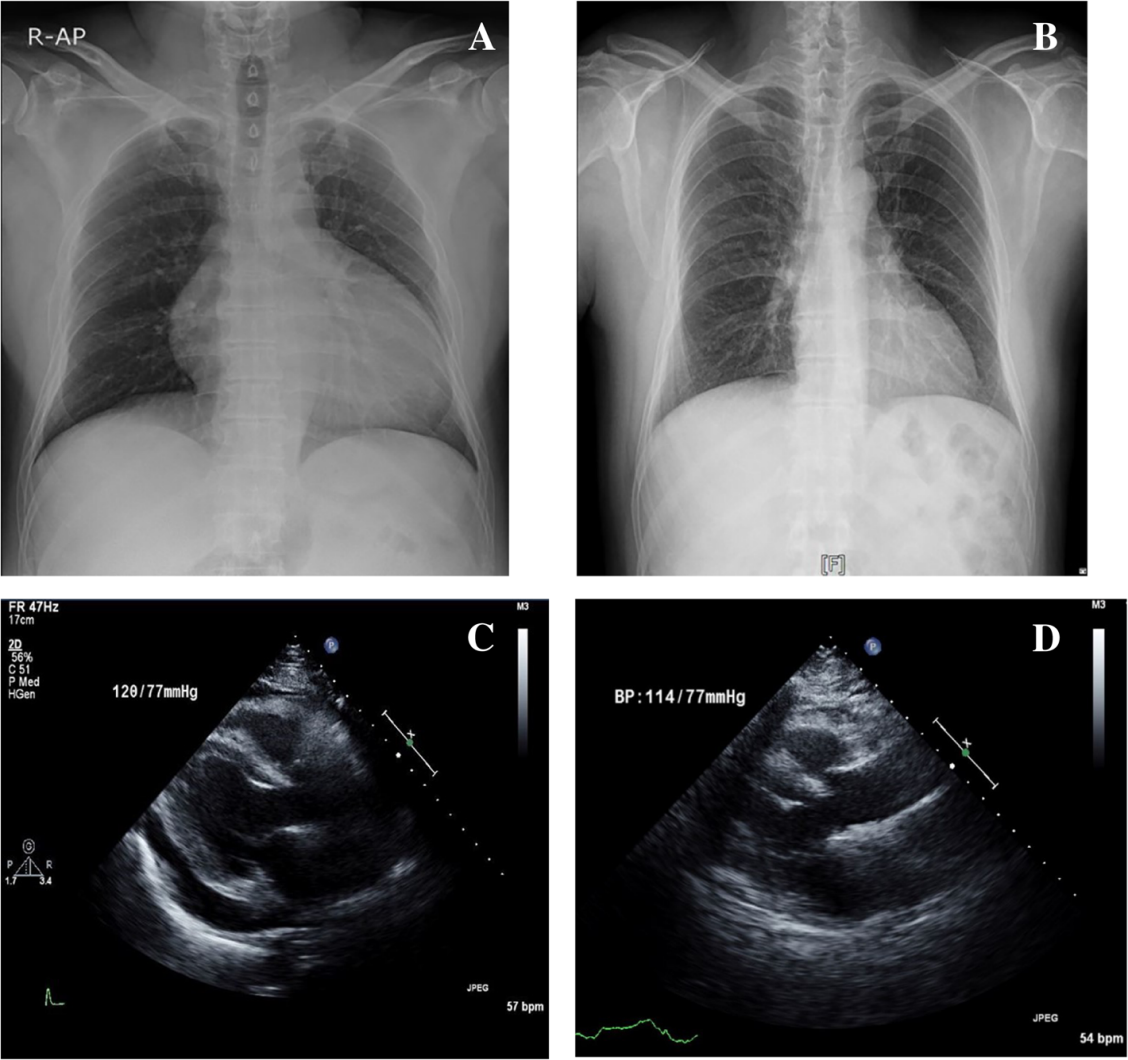
Image findings of the patient. Chest radiography shows cardiomegaly without pleural effusion at initial admission (**a**) and decreased cardiomegaly after discharge (**b**). Transthoracic echocardiography shows moderate pericardial effusion at initial admission (**c**) and completely recovered pericardial effusion at one-year later (**d**)

**Fig. 2:**
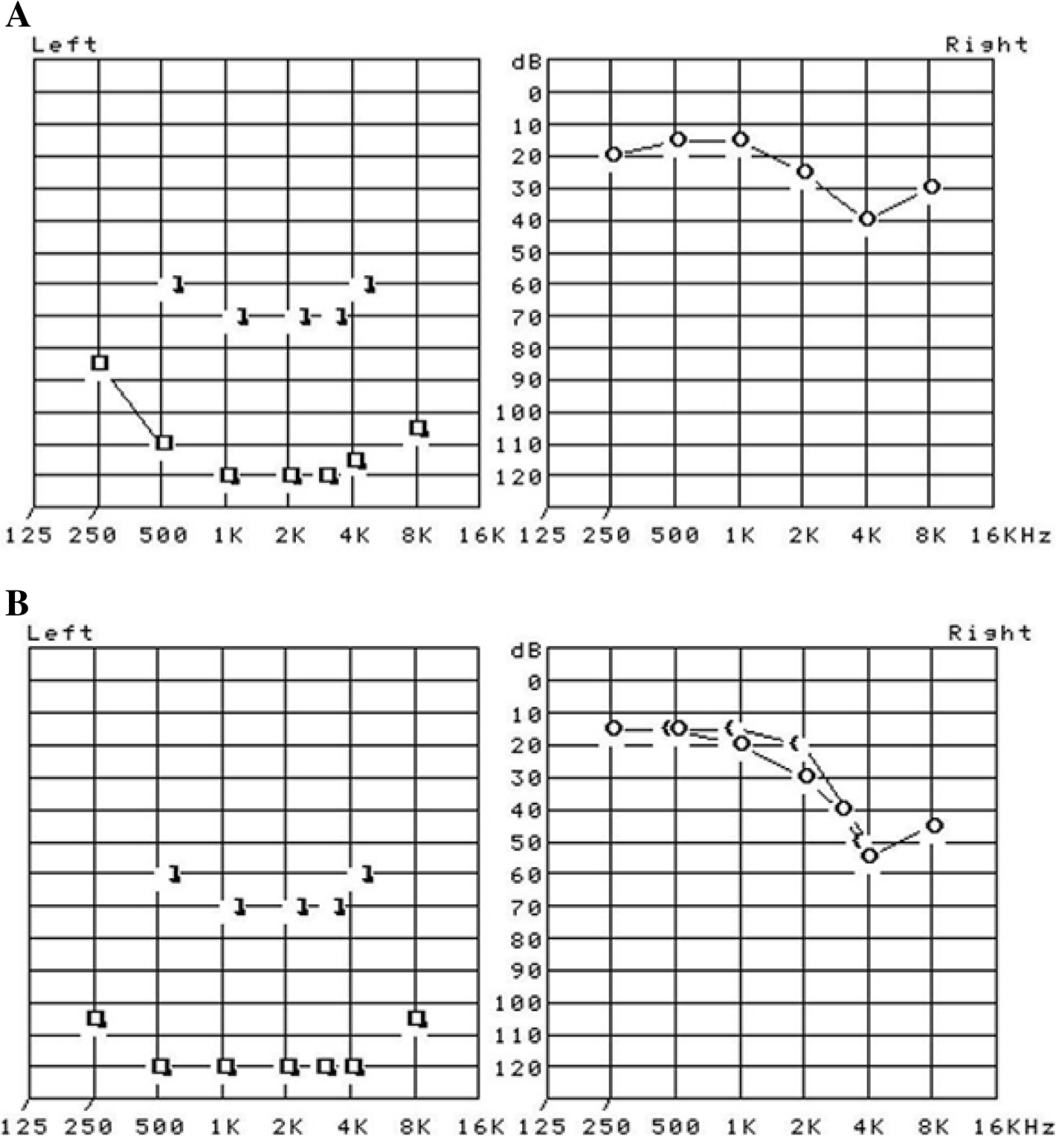
Pure tone audiometry displayed the presence of unilateral (left side) profound sensorineural hearing loss at initial diagnosis (**a**) and remained hearing loss at one-year after discharge (**b**)

**Fig. 3:**
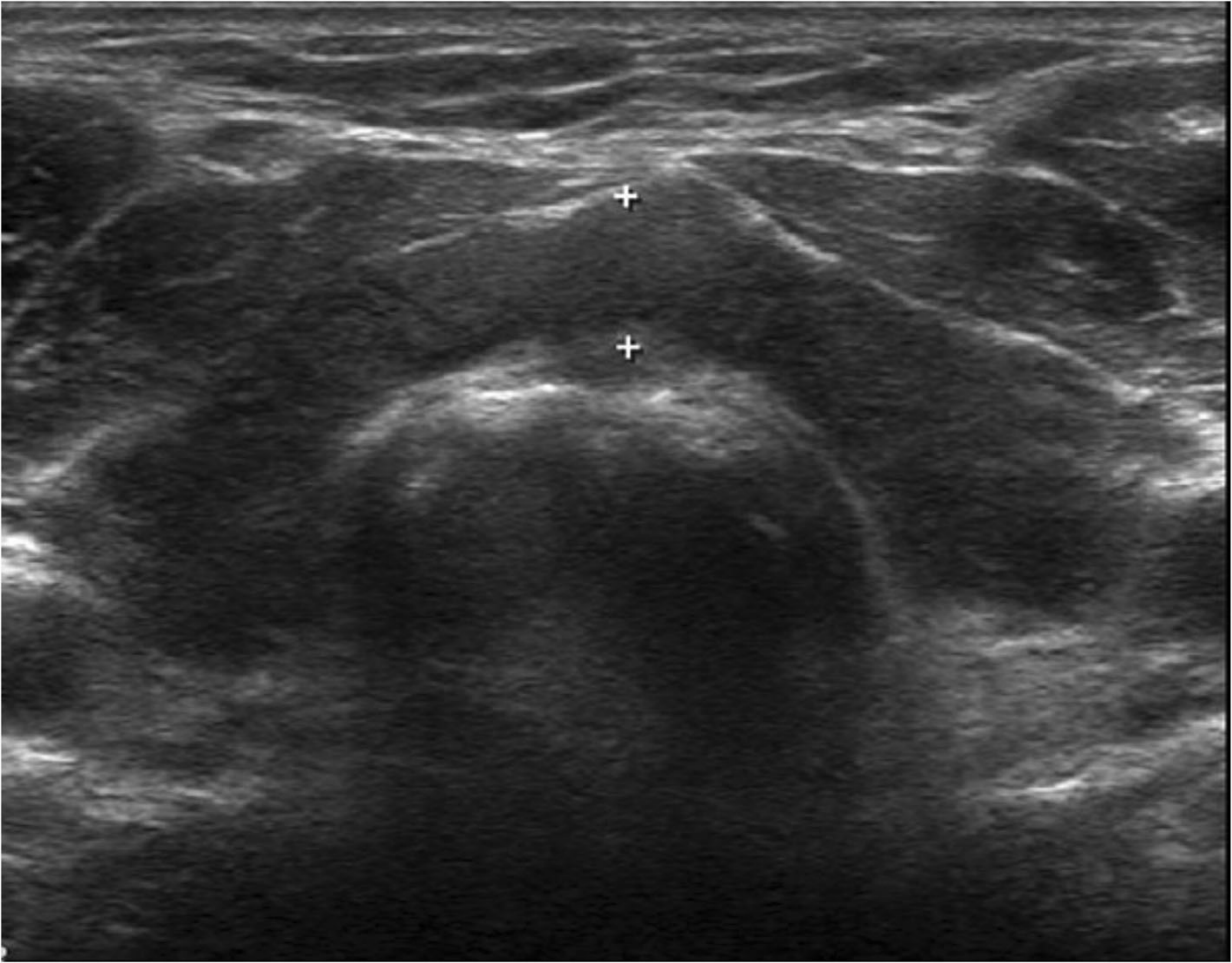
Transverse view of thyroid sonography revealed marked heterogenous echotexture of both thyroid glands

The initial results of laboratory tests (Table 1) were as follows: BUN 19.0 (7–23) mg/dL, creatinine 1.5 (0.7–1.4) mg/dL, creatine kinase 9300 (43–198) IU/L, lactate dehydrogenase 1876 (238–422) IU/L, myoglobulin 636 (28–72) ng/mL, alanine aminotransferase 357 (3–45) IU/L, aspartate aminotransferase 278 (3–45) IU/L, creatine kinase muscle-brain fraction (CK-MB) 52.07 (0–4.87) U/L, troponin I 0.057 (0–0.014) ng/mL, total cholesterol 222 (130–240) mg/dL, low density lipoprotein cholesterol (LDL-c) 136 (50–160) mg/dL, high density lipoprotein cholesterol (HDL-c) 74 (40–85) mg/dL, and triglycerides 127 (35–200) mg/dL. The results of thyroid function tests showed TSH >  100 (0.17–4.05) μIU/mL, fT4 0.32 (0.89–1.79) μIU/mL, and triiodothyronine (T3) 55.6 (78–182) ng/dL. On chest radiography, heart shadow was not blunted without pulmonary infiltration, and cardiomegaly was seen (cardiothoracic ratio, 66%; Fig. 1a). An electrocardiogram showed bradycardia with normal sinus rhythm and low QRS voltage, and transthoracic echocardiography revealed a moderate amount of pericardial effusion (posterior 15 mm) with preserved left ventricular systolic function (55–60%; Fig. 1c). On further investigation, pure tone audiometry revealed that the patient had profound unilateral sensorineural hearing loss (Fig. 2a), and thyroid sonography showed diffusely enlarged gland with a heterogeneous echotexture and decreased vascularity (Fig. 3); these findings are consistent with Hashimoto thyroiditis together with elevated thyroid peroxidase antibodies (> 2000 [0–5.61] IU/mL) and antithyroglobulin antibodies (9143.72 [0–4.11] IU/mL).

**Table 1:** Patient’s laboratory values

Variables	Reference range	Admission	Day 3	Day 7	Day 10	Day 20 (Outpatient clinic)
Hemoglobin, (g/dL)	13.1–17.2	12.0	12.9		12.2	12.2
WBC count, (× 10^3^/μL)	4.5–11.0	9.3	11.4		12.73	10.62
WBC Diff., neutrophils, (%)	40–75	64.7	88.8		70.0	66.9
BUN, (mg/dL)	7–23	19.0	15.2	12.9	17.0	22.8
Creatinine, (mg/dL)	0.7–1.4	1.5	1.34	1.11	1.07	1.16
Alkaline phosphatase, (U/L)	30–120	162	166			116
Alanine aminotransferase, (U/L)	3–45	357	270	273	138	47
Aspartate aminotransferase, (U/L)	3–45	278	221	147	49	27
Total cholesterol, (mg/dL)	130–240	222				
LDL cholesterol, (mg/dL)	50–160	136				
HDL cholesterol, (mg/dL)	40–85	74				
Triglycerides, (mg/dL)	35–200	127				
Creatine kinase, (IU/L)	43–198	9300	9936	1530	632	235
Lactate dehydrogenase, (IU/L)	238–422	1876	1818	1508	1333	457
Myoglobulin, (ng/mL)	28–72	636	428		61	47
CK-MB, (ng/mL)	0–4.87	52.07	42.60		11.24	5.94
Troponin I, (ng/mL)	0–0.014	0.057				
TSH, (μIU/mL)	0.17–4.05	> 100		53.65		48.17
fT4, (μIU/mL)	0.89–1.79	0.32		0.65		1.63
T3, (ng/dL)	78–182	55.6		71.6		86.2

Abbreviations: *BUN* blood urea nitrogen, *CK-MB* creatine kinase muscle-brain fraction, TSH thyroid stimulating hormone, *fT4* free thyroxine, *T3* triiodothyronine, *WBC* white blood cell, *LDL* low density lipoprotein, *HDL* high density lipoprotein

Upon admission, we started aggressive intravenous fluid resuscitation to treat rhabdomyolysis with acute kidney injury and levothyroxine replacement (oral, 150 mcg/day) to treat hypothyroidism. We also started systemic corticosteroids and intratympanic steroid injection to treat sensorineural hearing loss. The patient did not require pericardiocentesis because there was no ventricular dysfunction, and his vital signs were comparatively stable. Over the next several days, the patient’s clinical symptoms, such as shortness of breath, general weakness, and lower limb discomfort, improved substantially. On consecutive laboratory tests, creatine kinase as well as lactate dehydrogenase began to decrease on hospital day (HD) 3 and continued to decline to 1530 IU/L and 1508 IU/L on HD 7, respectively. Creatinine also started to decrease by 1.11 mg/dL on HD 7 accompanied by creatine kinase decline. Further thyroid function testing showed that his TSH was 53.65 μIU/mL and fT4 was 0.63 μIU/mL on HD 7. The patient was discharged home on HD 10 with instructions to continue levothyroxine (150 mcg/day, oral).

The patient was examined in the outpatient clinic 2 weeks after discharge. We observed recovered creatinine and creatinine kinase (Table 1) as well as improved cardiomegaly (Fig. 1b). Except for hearing loss, his clinical symptoms continued to improve, and he was compliant with levothyroxine treatment. His thyroid function was normalized at 5 months (TSH 0.10 μIU/mL, free T4 1.60 μIU/mL) and remained euthyroid status until one year after discharge (TSH 0.54 μIU/mL, free T4 1.52 μIU/mL). No pericardial effusion appeared on transthoracic echocardiography (Fig. 1d). Regarding hearing loss, there was slight improvement in follow-up pure tone audiometry after 1 year; nevertheless, profound unilateral hearing loss remained in this patient (Fig. 2b).

## Discussion and conclusions

Hypothyroidism is an endocrine disorder commonly encountered in real medical practice. Although clinical symptoms of hypothyroidism are generally diverse and nonspecific, it is seemingly straightforward to diagnose by means of simple and accurate laboratory tests. However, despite its simple and common diagnosis, it is difficult to speculate subsequent complications of hypothyroidism. Within this context, this report has shown simultaneous rare complications accompanying rhabdomyolysis, pericardial effusion, and sudden sensorineural hearing loss in the patient with untreated hypothyroidism. This case reminds physicians of the importance of timely diagnosis and proper treatment in hypothyroidism patients. Moreover, we should keep an eye on the critical complications, albeit uncommon, like rhabdomyolysis, pericardial effusion, and sudden sensorineural hearing loss in untreated hypothyroidism patients.

There have been several reports of hypothyroidism-induced rhabdomyolysis [8, 9, 11, 12], hypothyroidism-induced pericardial effusion [13, 14], and sensorineural hearing loss related to autoimmune thyroid disease [15–17] in recent decades. Zare-khormizi et al. presented the case of a patient with massive pericardial effusion and rhabdomyolysis secondary to untreated severe hypothyroidism [18]. However, to our best knowledge, ours is the first case report of simultaneous occurrence of rhabdomyolysis with acute kidney injury, moderate pericardial effusion, and sudden sensorineural healing loss in a patient with hypothyroidism.

Rhabdomyolysis, characterized by rapid skeletal muscle breakdown and release of intracellular muscle constituents into circulation, should be differentially diagnosed from other conditions [19]. Traumatic causes such as crush injury, multiple trauma, and immobilization as well as nontraumatic causes including extreme exercise, seizure, infection, and alcohol and drug abuse are well-known etiologies of rhabdomyolysis. High-dose statin treatment for hypercholesterolemia has recently been reported to cause rhabdomyolysis [20]. In the present case, the patient had been taking rosuvastatin 5 mg once daily for a year, which was not so potent dosage as to induce rhabdomyolysis; rosuvastatin is known as a more hydrophilic statin and is therefore less likely to enter the myocytes [21]. Moreover, no dosage changes were prescribed in this patient during treatment. He also had no history of trauma, other than drug abuse or alcohol intoxication. For these reasons, we deduced that the main cause of rhabdomyolysis was primary hypothyroidism. However, there is also a possibility that statin therapy and untreated hypothyroidism may synergistically enhance the risk of myopathy and rhabdomyolysis in patients as reported in the literature [22, 23]. The pathophysiology between rhabdomyolysis and hypothyroidism have not been clearly elucidated; however, thyroid hormone seems to be associated with glycogenolysis and mitochondrial oxidative metabolism in myocytes [24]. Furthermore, acute kidney injury in our case could be induced by rhabdomyolysis rather than hypothyroidism itself. Although hypothyroidism is known to affect renal impairment, increased creatinine was normalized in a short time after fluid resuscitation, consistent with creatine kinase decline, suggesting that decreased renal function was more likely associated with rhabdomyolysis than hypothyroidism.

Thyroid hormone is closely related to cardiac function, and pericardial effusion in patients with hypothyroidism is an infrequent manifestation. Decreased thyroid hormone is associated with protein extravasation and reduced lymphatic drainage, which leads to myxedema with fluid accumulation in the pericardial cavity [25, 26]. Cardiac tamponade has also been reported as a manifestation of severe hypothyroidism [27]. Therefore, it is reasonable to assess thyroid function in patients presenting with pericardial effusion or cardiac tamponade. Fortunately, in our patient, there was no evidence of cardiac tamponade and pericardial effusion decreased after levothyroxine treatment, without the need for pericardiocentesis.

Sudden sensorineural hearing loss is defined as acute onset of hearing loss of at least 30 dB occurring over a 72-h period and is nearly involving unilateral [28]. Identifiable causes are found in only 7–45% of patients with sudden sensorineural hearing loss. The most common causes are infectious diseases such as HIV, mycoplasma infection, or syphilis followed by otologic trauma or vascular, hematologic, or metabolic disorders such as hypothyroidism and diabetes mellitus [29]. Thyroid dysfunction can be found in patients presenting with sudden sensorineural hearing loss, with one report of a 15% rate of hypothyroidism [30]. However, metabolic disorders including hypothyroidism usually involve bilateral hearing loss [31]. Additional brain computed tomography (CT) and brain CT angiography conducted to find out other causes related to unilateral hearing loss did not detect any significant findings, and further brain magnetic resonance imaging could not be performed due to the patient’s refusal. Although we cannot completely exclude the possibility that the sensorineural hearing loss was not induced by hypothyroidism, it is more plausible to speculate that sudden sensorineural hearing loss was associated with untreated hypothyroidism in this patient who already have experienced hypothyroidism-related symptoms and complications like rhabdomyolysis and pericardial effusion. How to define the relationship between hypothyroidism and the auditory organ remains to be further investigated. Previous reports have suggested that thyroid autoantibodies, such as thyroglobulin antibody and thyroid peroxidase antibody, have a role in peripheral and central hearing organ dysfunction [17]. The prognosis of sudden sensorineural hearing loss is generally favorable; however, hearing loss in this case was not completely recovered even after glucocorticoid injection and levothyroxine treatment. Profound hearing loss at initial diagnosis across all frequencies is considered a risk factor for incomplete recovery [32].

In the current guidelines of the American Association of Clinical Endocrinologists and American Thyroid Association (AACE/ATA), thyroid function testing is recommended in patients with congestive heart failure, dementia, myopathy, and vitiligo to detect hypothyroidism [1]. Herein, we report for the first time a case of untreated hypothyroidism with concurrent rhabdomyolysis with acute kidney injury, pericardial effusion, and sudden sensorineural hearing loss. Our case report underlines the importance of appropriate diagnosis and treatment of hypothyroidism and suggests that it is reasonable to scrutinize thyroid function in patients with unexplained hearing loss and pericardial effusion as well as rhabdomyolysis.

## Abbreviations

BUN: Blood urea nitrogen
CK-MB: Creatine kinase muscle-brain fraction
fT4: Free thyroxine
HD: Hospital day
HIV: Human immunodeficiency virus
T3: Triiodothyronin
TSH: Thyroid stimulating hormone
